# Genetic variants associated with breast size also influence breast cancer risk

**DOI:** 10.1186/1471-2350-13-53

**Published:** 2012-06-30

**Authors:** Nicholas Eriksson, Geoffrey M Benton, Chuong B Do, Amy K Kiefer, Joanna L Mountain, David A Hinds, Uta Francke, Joyce Y Tung

**Affiliations:** 1 23andMe, Inc., Mountain View, CA 94043, USA

## Abstract

**Background:**

While some factors of breast morphology, such as density, are directly implicated in breast cancer, the relationship between breast size and cancer is less clear. Breast size is moderately heritable, yet the genetic variants leading to differences in breast size have not been identified.

**Methods:**

To investigate the genetic factors underlying breast size, we conducted a genome-wide association study (GWAS) of self-reported bra cup size, controlling for age, genetic ancestry, breast surgeries, pregnancy history and bra band size, in a cohort of 16,175 women of European ancestry.

**Results:**

We identified seven single-nucleotide polymorphisms (SNPs) significantly associated with breast size (*p*<5·10^−8^): rs7816345 near *ZNF703*, rs4849887 and (independently) rs17625845 flanking *INHBB*, rs12173570 near *ESR1*, rs7089814 in *ZNF365*, rs12371778 near *PTHLH*, and rs62314947 near *AREG*. Two of these seven SNPs are in linkage disequilibrium (LD) with SNPs associated with breast cancer (those near *ESR1* and *PTHLH*), and a third (*ZNF365*) is near, but not in LD with, a breast cancer SNP. The other three loci (*ZNF703*, *INHBB*, and *AREG*) have strong links to breast cancer, estrogen regulation, and breast development.

**Conclusions:**

These results provide insight into the genetic factors underlying normal breast development and show that some of these factors are shared with breast cancer. While these results do not directly support any possible epidemiological relationships between breast size and cancer, this study may contribute to a better understanding of the subtle interactions between breast morphology and breast cancer risk.

## Background

Breast morphology plays a complicated role in breast cancer risk. Most strikingly, mammographic density (the percent of non-fat breast tissue measured in a mammogram) is a risk factor for breast cancer [1]. Body weight, which is positively correlated with breast size, also has a complex relationship with breast cancer risk. Higher weight at younger ages decreases both premenopausal and postmenopausal breast cancer risk [2,3], while weight gain in adulthood increases postmenopausal breast cancer risk [4,5]. Breast asymmetry may also be associated with breast cancer risk [6]. The relationship between breast size and cancer is not entirely clear. Two studies have found that, for lean women, larger breast size is associated with a higher risk of breast cancer [7,8]. For example, Kusano et al. [7] found that among women with a BMI under 25, those with a cup size of D or larger had a 1.8 times higher risk of breast cancer than those with a cup size of A or smaller.

Genetic factors also play a role in breast cancer risk, with many genetic associations discovered to date. In contrast, there have been no genetic studies of breast size and only one GWAS of breast density [9]. Twin studies have shown that breast size is about 56% heritable, with only about a third of this heritability shared with the heritability of obesity [10]. However, to date, nothing is known about what genetic factors are associated with breast size. In this study, we examine the genetic factors underlying breast size through a GWAS of self-reported bra cup size in a cohort of 16,175 women of European ancestry. Of the seven significant associations we find with breast size, two are shared with breast cancer. In both cases, the same allele is linked to both increased breast size and increased breast cancer risk. A third breast size association is near a SNP associated with breast cancer risk and breast density, and the others are near genes with links to breast cancer and mammary development.

## Methods

### Subjects

Participants were drawn from the customer base of 23andMe, Inc., a consumer genetics company. This cohort has been described in detail previously [11,12]. All participants were female, of European ancestry, and no two shared more than 700 cM of DNA identical by descent (IBD, approximately the lower end of sharing between a pair of first cousins). IBD was calculated using the methods described in [13] and European individuals were selected as in [14]. Participants provided informed consent and participated in the research online, under a protocol approved by the external AAHRPP-accredited IRB, Ethical and Independent Review Services (E&I Review).

### Genotyping

Subjects were genotyped on one or more of three chips, two based on the Illumina HumanHap550+ BeadChip, the third based on the Illumina OmniExpress+ BeadChip. The platforms contained 586,916, 584,942, and 1,008,948 SNPs. Totals of 142, 4,764, and 11,890 participants were genotyped on the platforms, respectively. A total of 621 individuals were genotyped on multiple chips. For all participants, we imputed genotypes against the August 2010 release of the 1000 Genomes reference haplotypes [15]. First, we used Beagle 3.3.1 [16] to phase batches of 8000–9000 individuals across chromosomal segments of no more than 10,000 genotyped SNPs, with overlaps of 100 SNPs. Individuals were grouped by genotyping array before this phasing, which was performed on the entire 23andMe cohort. Across this larger cohort, a total of ten batches were run, four consisting of individuals typed on the first two platforms and the other six of individuals typed on the third platform. Before phasing, we excluded SNPs with minor allele frequency under 0.001, *p*-value for Hardy-Weinberg equilibrium under 10^-20^, or call rate under 95%. The threshold for the Hardy-Weinberg test is smaller than usual because the *p*-values were computed in a sample of nearly 100,000 European individuals; our threshold of 10^-20^ corresponds roughly to a threshold of 10^-4^ for a sample size of 20,000 individuals. We also excluded SNPs with large allele frequency discrepancies compared to the 1000 Genomes reference data. We then assembled full phased chromosomes by matching the phase of haplotypes across the overlapping segments. We imputed each batch against the European subset of 1000 Genomes haplotypes with Minimac [17], using 5 rounds and 200 states for parameter estimation. A total of 11,914,767 SNPs were imputed. Of these, 7,422,970 met our thresholds of 0.001 minor allele frequency and *r*^2^ of at least 0.5 (averaged across batches).

Imputation quality was slightly higher overall for the batches using the denser platform (average *r*^2^ of 0.91 versus 0.87). In the tables, we thus report the *r*^2^ separately for the first four batches and the last six batches (denoted $r_{V2}^{2}$ and $r_{V3}^{2}$, respectively). The overall *r*^2^ can be calculated as $r^{2}=0.45\cdot r_{V2}^{2}+0.55\cdot r_{V3}^{2}$. *p*-values for Hardy-Weinberg equilibrium were calculated using the test from [18] on dosages rounded to the nearest integer (set to NA if the dosage was more than 0.25 away from an integer). Positions and alleles are given relative to the positive strand of build 37 of the human genome.

### Phenotype data collection

All participants reported bra cup size and bra band size as part of an online body morphology questionnaire. Participants selected a cup size from nine categories and entered band size as an integer. Those entering a band size of over 70 were assumed to be using centimeters and were removed from analysis.

Subsets of participants also reported other phenotypes. As covariates in the analysis, we included the projections onto the first five principal components of genetic ancestry as well as age, bra band size (in inches), and indicator variables for breast augmentation surgery, breast reduction surgery, mastectomy, past pregnancy, and current pregnancy or breastfeeding. Out of the 16,175 participants, all but 3 reported age, over 15,000 reported bra band size, about 12,000 reported breast surgery status (augmentation, reduction, mastectomy, or none), about 6,000 reported if they had ever been pregnant, and 4,000 reported if they were currently pregnant or breastfeeding.

Band size was used as a covariate instead of BMI because while almost every participant reported band size, only about half reported BMI. Band size has previously been used as a proxy for BMI in breast size research [8]. The correlation between BMI and band size in our sample was over 0.5. Furthermore, although bra size is easy to report, it is not a perfect proxy for actual breast volume. There is evidence that controlling for bra band size improves the correlation between cup size and breast volume [19].

### Statistical analysis

Bra size was coded from 0 to 9, corresponding to the categories: Smaller than AAA, AAA, AA, A, B, C, D, DD, DDD, and Larger than DDD, respectively. Mean size was 4.99 (just under a “C” cup) and the standard deviation was 1.45. Genotypes were coded as dosages from 0–2, corresponding to the estimated number of copies of the minor allele present. *p*-values for SNPs were calculated using likelihood ratio tests for linear regressions.

The principal component analysis was done as described before [11]. Individuals who were missing some of the covariates were imputed to the average value among those who did provide data. Average values for age and bra band size were 47.4 years and 36.2 inches, respectively. The percentages answering “yes” to other covariates were: breast augmentation, 5%; breast reduction, 3%; mastectomy, 1.7%; ever pregnant, 80%; and currently pregnant or breastfeeding, 3%.

On average, those reporting augmentation reported 0.5 size smaller breasts, reduction 1 size bigger, mastectomy 0.5 size smaller, and ever pregnant 0.1 size bigger. For every inch of band size, cup size was reported to be 0.1 sizes bigger on average. Current pregnancy did not significantly influence reported cup size. Some participants commented that they reported their pre-surgery or pre-pregnancy breast size. As those reporting augmentation also reported smaller breasts on average, it may be the case that many participants reported pre-surgery size. If that is the case generally, including these covariates just led to decreased power. We looked at effect sizes for the SNPs in Table 1 with and without including the covariates beyond age and PCs and saw no significant differences in effect sizes.

**Table 1:** **Index SNPs for regions under*p*= 10**^**-6**^

										
**SNP**	**Chr**	**Position**	**Gene**	**Allele**	**MAF**	**HWE**	$r_{V2}^{2}$	$r_{V3}^{2}$	*p*-value	*β*
rs7816345	8	36846109	*ZNF703*	C/T	0.194	0.08	1.00	1.00	1.64·1^0−14^	-0.151 (-0.189 – -0.112)
rs4849887	2	121245122	*INHBB*	C/T	0.113	0.97	1.00	0.94	3.31·1^0−11^	0.166 (0.117 – 0.214)
rs17625845	2	121089731	*INHBB*	T/C	0.205	0.07	0.72	0.99	4.7·1^0−10^	0.125 (0.086 – 0.164)
rs12173570	6	151957714	*ESR1*	C/T	0.101	0.82	0.98	0.99	5.58·1^0−11^	0.171 (0.120 – 0.222)
rs7089814	10	64187564	*ZNF365*	T/C	0.375	0.97	0.94	0.98	3.3·1^0−9^	0.096 (0.064 – 0.128)
rs12371778	12	28156081	*PTHLH*	C/G	0.091	0.72	0.85	0.87	1.03·1^0−8^	-0.162 (-0.217 – -0.106)
rs62314947	4	75502487	*AREG*	C/T	0.281	0.31	0.73	0.91	4.79·1^0−8^	-0.101 (-0.137 – -0.065)
rs4820792	22	29161007	*CHEK2*	C/T	0.180	0.01	0.95	0.97	4.17·1^0−7^	0.105 (0.065 – 0.146)
chr22:40779964	22	40779964	*MKL1*	G/A	0.056	0.05	0.75	0.78	5.47·1^0−7^	-0.187 (-0.261 – -0.114)
rs61280460	14	94796184	*SERPINA6*	A/T	0.199	0.29	1.00	1.00	8.3·1^0−7^	-0.095 (-0.132 – -0.057)

The index SNP is defined as the SNP with the smallest *p*-value within a region; or the SNP with the smallest *p*-value in the conditional analysis. The listed gene is our postulated candidate gene near the SNP. For *INHBB*, conditional analysis revealed two independent SNPs in the region. Alleles are listed as major/minor (in Europeans). MAF is the frequency of the minor allele in Europeans and $r_{V2}^{2}$ and $r_{V3}^{2}$ are the estimated imputation accuracies on the two genotyping platforms. HWE is the *p*-value for Hardy-Weinberg equilibrium (calculated on rounded dosages). The coefficient *β*refers to the average change in breast size (in units of cup size) per copy of the minor allele.

We performed conditional analyses within each genome-wide significant region to search for SNPs with independent effects. We analyzed each SNP within 500kb from each index SNP, adding the index SNP to the covariates for this analysis (and continuing the process iteratively if any SNPs remained with a *p*-value of 10^-6^).

## Results and discussion

**Figure 1:**
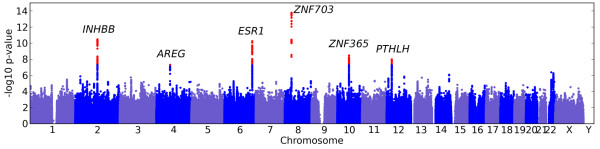
**Manhattan plot of association with breast size.**$-\underset{10}{\text{log}}p$-values across all SNPs tested. SNPs shown in red are genome-wide significant (*p*<5·1^0−8^). Regions are named with the postulated candidate gene.

We identified six regions containing SNPs significantly associated with breast size (using a threshold of 5·1^0−8^ for genome-wide significance), see Figure 1. The genomic control inflation factor for this study was 1.047 (see Additional file 1 for the quantile-quantile plot).

**Figure 2:**
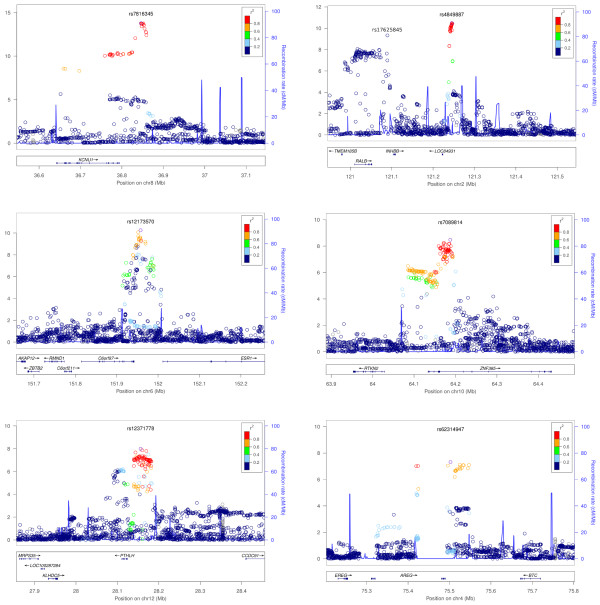
**Associations with breast size in six regions with genome-wide significant SNPs.** Colors depict the squared correlation (*r*^2^) of each SNP with the most associated SNP (which is shown in purple). Gray indicates SNPs for which *r*^2^ information was missing. For the plot labeled with rs7816345, the gene *ZNF703* lies about 400kb outside the region displayed.

The SNPs with the smallest *p*-values in these regions are rs7816345 in 8p12 (a region that is amplified in breast tumors and contains the breast cancer oncogene *ZNF703* (zinc finger protein 703)), rs4849887 near *INHBB* (inhibin, beta B), rs12173570 near *ESR1* (estrogen receptor 1), rs7089814 in *ZNF365* (zinc finger protein 365), rs12371778 near *PTHLH* (parathyroid hormone-like hormone), and rs62314947 near *AREG* (amphiregulin); see Table 1 for details and Figure 2 for plots of *p*-values in these regions. All SNPs with *p*-values under 10^-4^ are shown in Additional file 2.

First, rs7816345 (*p*=1.64·1^0−13^), lies within the 8p12 region commonly amplified in the luminal B subtype of estrogen receptor (ER) positive breast cancers that have a poor clinical outcome [20]. *ZNF703*, the only gene in the minimal amplified region, is the likely oncogene driving this amplification [21]. *ZNF703* is up-regulated by estrogen and is a co-factor for a nuclear repressor complex that plays a role in the regulation of ER activity. It has also been implicated in the regulation of cell proliferation, and its overexpression leads to an increase in breast cancer stem cells [21,22]. Interestingly, *ZNF703* exerts a downstream effect on the TGF beta signaling pathway [22] and also cooperates with a form of p53 [23].

rs4849887 (*p*=3.31·1^0−11^) lies 140kb downstream of the closest gene, *INHBB*. *INHBB* is a subunit of both inhibin and activin, hormones in the TGF beta superfamily that are important for many endocrine functions. While both *INHBA* (inhibin, beta A) and *INHBB* are expressed in normal breast tissue, only *INHBB* is up-regulated by estrogen [24]. Activin A (an inhibin beta A homodimer) is more highly expressed in breast cancer [25], though *INHBB* has been implicated in the carcinogenesis of non-endometrial uterine cancer [26]. *INHBB* is also highly expressed in fat cells, and its expression is reduced by weight loss [27]. A conditional analysis in this region, controlling for rs4849887, revealed a second, independent association with breast size: rs17625845 (*p*-value 4.7·1^0−10^ in the initial analysis and 5.85·1^0−10^controlling for rs4849887). This SNP is located upstream of *INHBB*.

The next SNP association with breast size is rs12173570, located near *ESR1*, (*p*=5.58·1^0−11^). rs12173570 is in LD with rs9397435 (^*r*2^=0.56), which is also associated with breast size (*p*=1.15·1^0−9^). rs9397435 has previously been associated with breast cancer in European, Asian, and African populations and affects the expression of *ESR1*[28]. The G allele of rs9397435 corresponds to larger breast size, increased cancer risk, and increased expression. *ESR1* is of great importance in normal breast development and cancer, and there is evidence that rs2046210 (which is in LD with rs9397435 in Asian populations (^*r*2^=0.73) but less so in European populations (^*r*2^=0.13)) may be associated with breast density [9].

The fourth region associated with breast size is centered around *ZNF365* (rs7089814, *p*=3.30·1^0−9^). This SNP lies in an intron of *ZNF365*, about 90kb away from rs10995190. rs10995190 has been associated with both breast cancer [29] and breast density [9]. rs7089814 and rs10995190 are not in LD (^*r*2^=0.035), and there is some evidence that rs10995190 is associated with breast size independently from rs7089814 (*p*=8.5·1^0−4^ initially (Table 2) and 1.7·1^0−3^after correction for rs7089814).

**Table 2:** Association with breast size for SNPs previously associated with breast cancer


**SNP**	**Chr**	**Position**	**Gene**	**Allele**	**MAF**	**HWE**	$r_{V2}^{2}$	$r_{V3}^{2}$	***p*-value**	*β*
rs9397435	6	151951220	*ESR1*	A/G	0.080	0.34	0.93	0.94	1.14·10^−9^	0.183 (0.124 – 0.242)
rs2046210	6	151948366	*ESR1*	G/A	0.355	0.89	0.97	1.00	1.33·1^0−8^	0.092 (0.060 – 0.124)
rs10771399	12	28155080	*PTHLH*	A/G	0.111	0.50	0.94	0.99	5.87·1^0−8^	-0.133 (-0.181 – -0.085)
rs10995190	10	64278682	*ZNF365*	G/A	0.155	0.15	1.00	1.00	0.000857	-0.071 (-0.113 – -0.029)
rs3803662	16	52586341	*TOX3*	G/A	0.276	0.16	1.00	1.00	0.0225	0.040 (0.006 – 0.074)
rs3817198	11	1909006	*LSP1*	T/C	0.324	0.13	1.00	1.00	0.0632	0.031 (-0.002 – 0.063)
rs2823093	21	16520832	21q21	G/A	0.273	0.23	1.00	1.00	0.133	0.026 (-0.008 – 0.060)
rs16886165	5	56023083	*MAP3K1*	T/G	0.158	0.95	1.00	1.00	0.137	-0.031 (-0.073 – 0.010)
rs981782	5	45285718	5p12	A/C	0.455	0.03	1.00	1.00	0.146	0.022 (-0.008 – 0.053)
rs10941679	5	44706498	5p12	A/G	0.241	0.06	0.66	1.00	0.158	0.027 (-0.010 – 0.064)
rs999737	14	69034682	*RAD51L1*	C/T	0.226	0.69	1.00	1.00	0.182	-0.025 (-0.061 – 0.012)
rs889312	5	56031884	*MAP3K1*	A/C	0.286	0.27	1.00	1.00	0.221	-0.021 (-0.054 – 0.013)
rs1219648	10	123346190	*FGFR2*	A/G	0.402	0.65	1.00	1.00	0.223	0.019 (-0.012 – 0.050)
rs2981582	10	123352317	*FGFR2*	G/A	0.397	0.79	1.00	1.00	0.304	0.016 (-0.015 – 0.047)
rs1562430	8	128387852	8q24.21	T/C	0.423	0.03	1.00	1.00	0.308	-0.016 (-0.046 – 0.015)
rs6504950	17	53056471	17q23.2	G/A	0.274	0.89	1.00	1.00	0.317	0.017 (-0.017 – 0.051)
rs7222197	17	53047499	*STXBP4*	G/A	0.274	0.91	1.00	1.00	0.317	0.017 (-0.017 – 0.051)
rs2380205	10	5886734	*ANKRD16*	C/T	0.428	0.10	1.00	1.00	0.327	0.015 (-0.015 – 0.046)
rs2981579	10	123337335	*FGFR2*	G/A	0.416	0.26	1.00	1.00	0.339	0.015 (-0.016 – 0.046)
rs704010	10	80841148	*ZMIZ1*	C/T	0.379	0.32	1.00	1.00	0.418	0.013 (-0.018 – 0.044)
rs1292011	12	115836522	12q24	A/G	0.422	0.70	0.99	1.00	0.428	-0.012 (-0.043 – 0.018)
rs1045485	2	202149589	*CASP8*	G/C	0.123	0.92	1.00	1.00	0.472	-0.017 (-0.062 – 0.029)
rs614367	11	69328764	*MYEOV*	C/T	0.149	0.42	0.98	0.99	0.525	-0.014 (-0.057 – 0.029)
rs1011970	9	22062134	*CDKN2A*	G/T	0.171	0.12	1.00	1.00	0.589	0.011 (-0.029 – 0.051)
rs4973768	3	27416013	3p24	C/T	0.485	0.26	1.00	1.00	0.591	0.008 (-0.022 – 0.038)
rs13387042	2	217905832	2q35	A/G	0.476	0.60	1.00	1.00	0.613	0.008 (-0.023 – 0.038)
rs11249433	1	121280613	1p11.2	A/G	0.424	0.05	0.94	0.95	0.744	-0.005 (-0.037 – 0.026)
rs13281615	8	128355618	8q24.21	A/G	0.416	0.06	1.00	1.00	0.976	0.000 (-0.030 – 0.031)

Columns are as in Table 1.

rs12371778, near *PTHLH*, is associated with breast size (*p*=1.03·1^0−8^), and is in LD (^*r*2^=0.82) with rs10771399, which has previously been associated with breast cancer [30]. The A allele of rs10771399 is the risk allele for breast cancer and corresponds to the C allele of rs12371778, which is associated with larger breast size. *PTHLH* encodes a member of the parathyroid hormone family that plays a key role in embryonic mammary development [31] as well as lactation [32].

Finally, rs62314947, *p*=4.79·1^0−8^, near *AREG*, barely falls under our threshold for genome-wide significance. Amphiregulin is related to the epidermal growth factor and TGF alpha families. It mediates ER function in mammary development [33,34].

Three SNPs have *p*-values under 10^-6^ but are not genome-wide significant (Additional file 3). First, rs4820792 (*p*=4.17·1^0−7^) lies 25kb upstream of *CHEK2* (checkpoint kinase 2), which is involved in the response to DNA damage. The 1100delC mutation in *CHEK2* is strongly associated with breast cancer; however, 1100delC and rs4820792 are not in LD. Next, chr22:40779964 (*p*=5.47·1^0−7^) lies in *SGSM3* (small G protein signaling modulator 3), near *MKL1*, megakaryoblastic leukemia (translocation) 1. Finally, rs61280460 (*p*=8.30·1^0−7^) lies near *SERPINA6* (serpin peptidase inhibitor, clade A (alpha-1 antiproteinase, antitrypsin), member 6).

Motivated by the above overlaps between breast cancer and breast size SNPs, we analyzed 29 SNPs that have previously been associated with breast cancer (from [29,30] and the supplement of [9]) for association with breast size in our data (Table 2). Of these 29 SNPs, only four were significant after correcting for 29 tests; these are the SNPs mentioned above near *ESR1* (two SNPs), *PTHLH*, and *ZNF365*.

There is a strong relationship in our data between BMI and breast size—each additional BMI unit corresponds to an increase of about 0.1 cup sizes on average. However, the SNPs in Table 1 are not in LD with any variants previously associated with BMI [35]; this is expected due to the inclusion of bra band size (which is correlated with BMI) as a covariate. Furthermore, even if we did not control for BMI, the strongest associations with BMI (e.g., rs1558902 near *FTO*) have effects of about 0.4 BMI units per allele. This would correspond to an expected *β*of about 0.04 for breast size for these SNPs, which is below the effect sizes we are powered to detect here. Indeed, if bra band size is not included as a covariate, rs1558902 has an estimated *β* of 0.07 (95% CI: 0.04 – 0.10) for breast size and *p*-value of 8·1^0−6^as compared to *β*of 0.04 (95% CI: 0.01 – 0.07) with bra band size included.

The covariates included in the analysis explain about 9.7% of the variance in breast size in our data; including the 7 SNPs in Table 1 that are genome-wide significant increases this to 10.9%. We used these 7 SNPs to compute a genetic propensity score for breast size by counting the number of alleles associated with larger size that each participant carried. The average cup size among women in the top 5% of this score (women carrying 9 or more of the 14 possible “large” alleles) was 0.83 cup sizes bigger (5.39 versus 4.56) than the average cup size among women in the bottom 5% of this score (women carrying 4 or fewer “large” alleles).

We note that the estimation of breast volume via self-reported bra size is likely to be far from perfect. Thus, it would be interesting to see what effects the SNPs found here would have in a more exactly phenotyped population. Likewise, many of the SNPs reported here were only imputed and not directly typed. While the estimated *r*^2^ values are generally quite high, indicating good imputation quality, ideally these SNPs would be directly typed in a replication cohort.

## Conclusions

Surprisingly, this GWAS demonstrates that variants in some of the same gene regions are involved in both breast size and breast cancer. We have shown that two SNPs (rs12173570 near *ESR1* and rs12371778 near *PTHLH*) are associated with breast size; these two SNPs have previously been associated with breast cancer risk. A third SNP previously associated with breast cancer, rs10995190 in *ZNF365*, shows a possible association with breast size; in addition, we find a second SNP in the same gene (rs7089814) that is significantly associated with breast size. It should be noted that the shared relationships between breast size and breast cancer at these three regions are not strong enough to account for the possible epidemiological connection that has been reported elsewhere between breast size and breast cancer.

The other associations we have found are near genes involved in other aspects of breast cancer and estrogen pathways. We find two independent associations with size flanking the gene *INHBB*, which has connections to estrogen regulation, obesity, and uterine cancer. Of the final two associations, one lies in the 8p12 region amplified in breast tumors (near *ZNF703*) and the last lies near *AREG*, which plays a role mediating estrogen function in breast development.

Our results identify genetic variants that have an effect on both breast cancer and natural variation in breast size. While the precise relationships between breast size, density, obesity and breast cancer remain difficult to untangle, understanding the biology behind these developmental processes may be important in understanding breast cancer and may aid in the development of novel screening tools.

## Abbreviations

AAHRPP: Association for the Accreditation of Human Research Protection Programs; AREG amphiregulin; BMI: body mass index; ER: estrogen receptor; ESR1: estrogen receptor 1; GWAS: genome-wide association study; HWE: Hardy-Weinberg equilibrium; INHBA: inhibin, beta A; INHBB: inhibin, beta B; IRB: institutional review board; LD: linkage disequilibrium; MAF: minor allele frequency; p53, protein 53; PTHLH: parathyroid hormone-like hormone; SNP: single nucleotide polymorphism; TGF: transforming growth factor; ZNF365; ZNF703: zinc finger protein 703.

## Competing interests

The authors of this paper are 23andMe employees and own stock options in the company.

## Author’s contributions

NE, GB, CBD, AKK, JLM, DAH, UF, and JYT conceived and designed the experiments. NE and CBD analyzed the data. NE drafted the manuscript with contributions from all other authors.

## Pre-publication history

The pre-publication history for this paper can be accessed here:

http://www.biomedcentral.com/1471-2350/13/53/prepub

## Supplementary Material

Additional file 1 **Quantile-quantile plot of association with breast size.** Observed *p*-values versus theoretical *p*-values under the null hypothesis of no association. The genomic control inflation factor for the study was 1.047 and is indicated by the red line; approximate 95% confidence intervals are given by the blue curves. Click here for file

Additional file 2 **All SNPs with*p*<1**0^**−4**^**for breast size.** Alleles are listed as major/minor. MAF is the frequency of the minor allele in Europeans, and $r_{V2}^{2}$ and $r_{V3}^{2}$ are the estimated imputation accuracies on the two genotyping platforms. Click here for file

Additional file 3 **Associations with breast size in three regions with suggestive SNPs.** Colors depict the squared correlation (*r*^2^) of each SNP with the most associated SNP (which is shown in purple). Gray indicates SNPs for which *r*^2^ information was missing. Click here for file
