# Supplementary figures and images for: Genetic variants associated with breast size also influence breast cancer risk
Source: BMC Med Genet. 2012 Jun 30;13:53. doi: 10.1186/1471-2350-13-53 (PMC3483246; doi:10.1186/1471-2350-13-53)

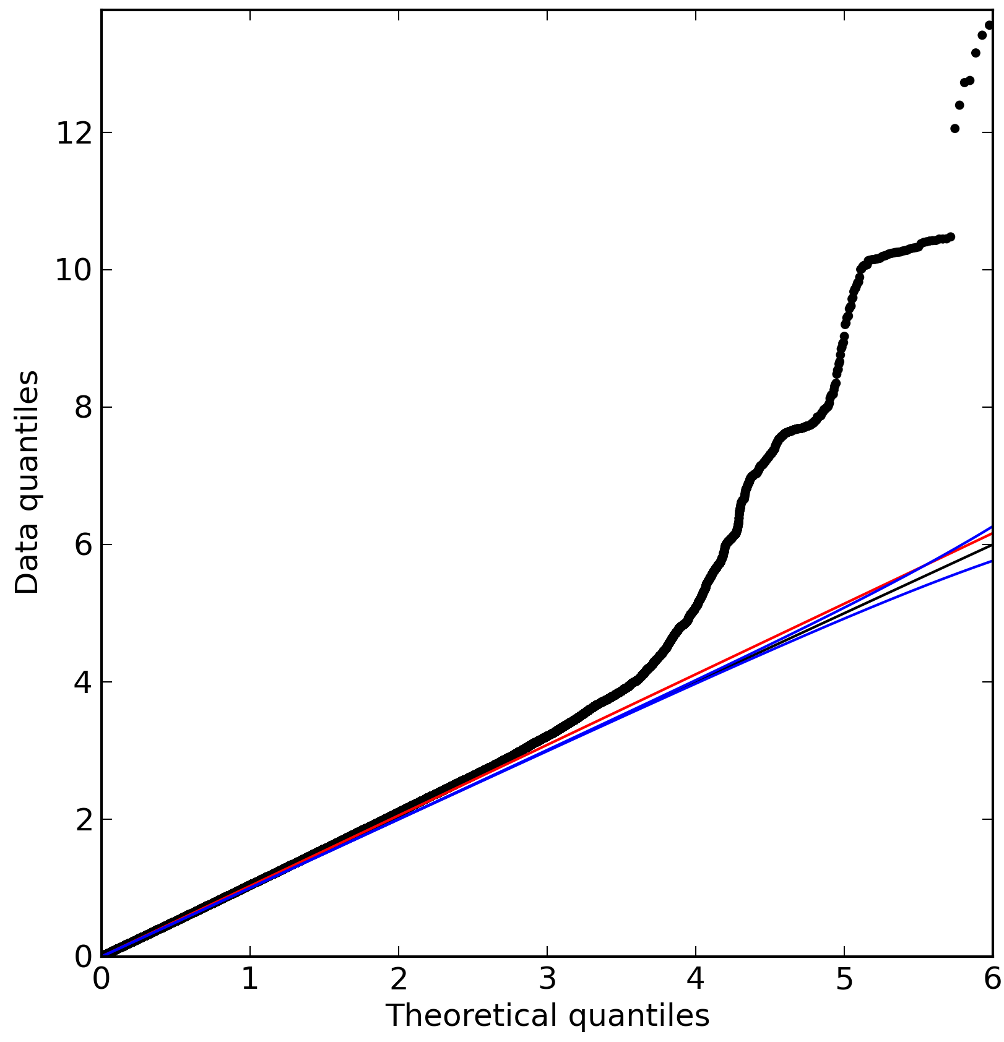

Supplement: Additional file 1 — Quantile-quantile plot of association with breast size. Observed p-values versus theoretical p-values under the null hypothesis of no association. The genomic control inflation factor for the study was 1.047 and is indicated by the red line; approximate 95% confidence intervals are given by the blue curves. [file 1471-2350-13-53-S1.tiff]

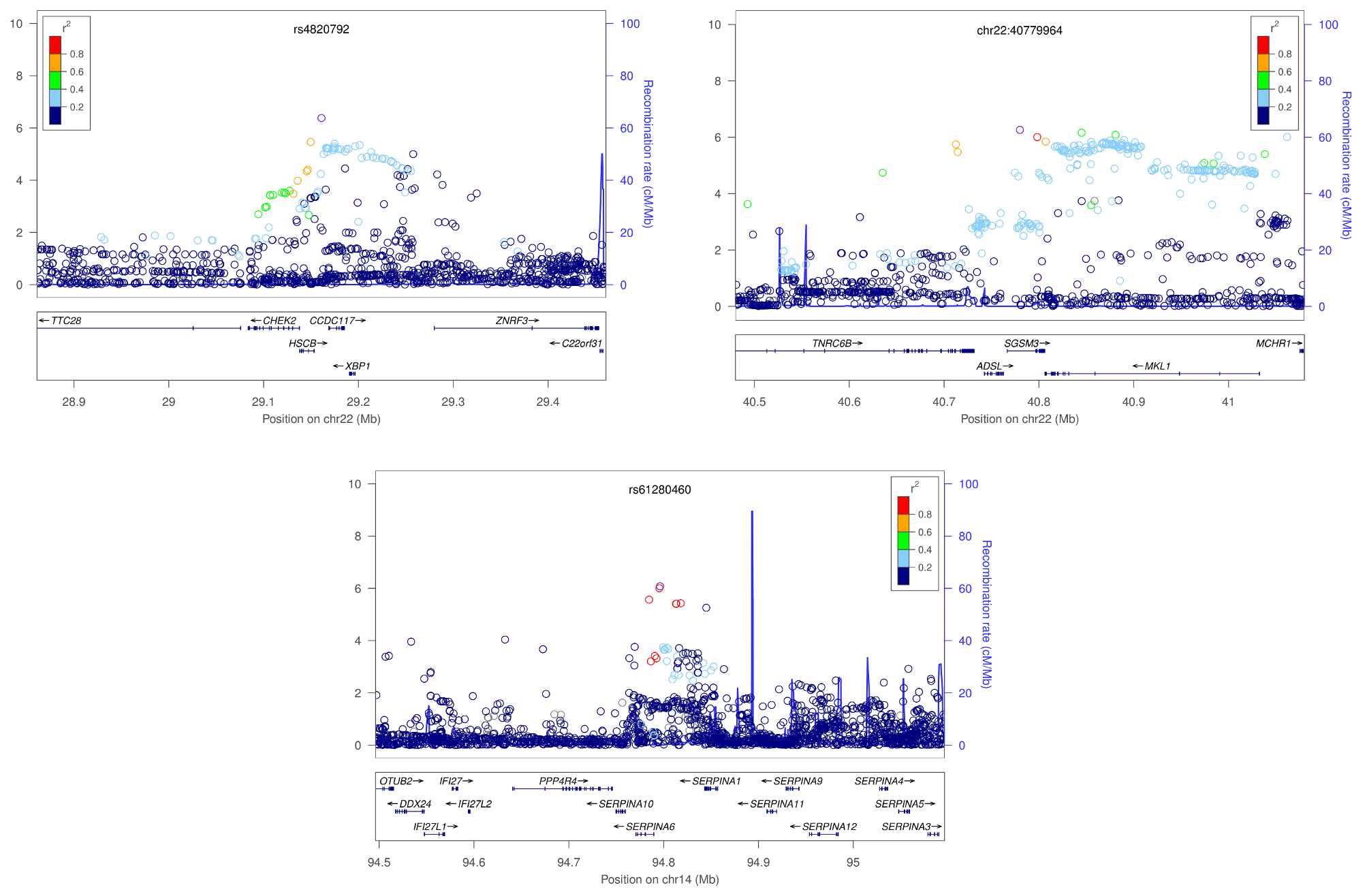

Supplement: Additional file 3 — Associations with breast size in three regions with suggestive SNPs. Colors depict the squared correlation (r2) of each SNP with the most associated SNP (which is shown in purple). Gray indicates SNPs for which r2 information was missing. [file 1471-2350-13-53-S3.png]
